# An Integrative -omics Approach to Identify Functional Sub-Networks in Human Colorectal Cancer

**DOI:** 10.1371/journal.pcbi.1000639

**Published:** 2010-01-15

**Authors:** Rod K. Nibbe, Mehmet Koyutürk, Mark R. Chance

**Affiliations:** 1 Center for Proteomics & Bioinformatics, Case Western Reserve University, Cleveland, Ohio, United States of America; 2 Department of Pharmacology, Case Western Reserve University, Cleveland, Ohio, United States of America; 3 Department of Electrical Engineering & Computer Science, Case Western Reserve University, Cleveland, Ohio, United States of America; 4 Department of Physiology & Biophysics, Case Western Reserve University, Cleveland, Ohio, United States of America; University of Illinois at Urbana-Champaign, United States of America

## Abstract

Emerging evidence indicates that gene products implicated in human cancers often cluster together in “hot spots” in protein-protein interaction (PPI) networks. Additionally, small sub-networks within PPI networks that demonstrate synergistic differential expression with respect to tumorigenic phenotypes were recently shown to be more accurate classifiers of disease progression when compared to single targets identified by traditional approaches. However, many of these studies rely exclusively on mRNA expression data, a useful but limited measure of cellular activity. Proteomic profiling experiments provide information at the post-translational level, yet they generally screen only a limited fraction of the proteome. Here, we demonstrate that integration of these complementary data sources with a “proteomics-first” approach can enhance the discovery of candidate sub-networks in cancer that are well-suited for mechanistic validation in disease. We propose that small changes in the mRNA expression of multiple genes in the neighborhood of a protein-hub can be synergistically associated with significant changes in the activity of that protein and its network neighbors. Further, we hypothesize that proteomic targets with significant fold change between phenotype and control may be used to “seed” a search for small PPI sub-networks that are functionally associated with these targets. To test this hypothesis, we select proteomic targets having significant expression changes in human colorectal cancer (CRC) from two independent 2-D gel-based screens. Then, we use random walk based models of network crosstalk and develop novel reference models to identify sub-networks that are statistically significant in terms of their functional association with these proteomic targets. Subsequently, using an information-theoretic measure, we evaluate synergistic changes in the activity of identified sub-networks based on genome-wide screens of mRNA expression in CRC. Cross-classification experiments to predict disease class show excellent performance using only a few sub-networks, underwriting the strength of the proposed approach in discovering relevant and reproducible sub-networks.

## Author Summary

Intensive research on cancer has led to an understanding of many individual genes that may be important for the initiation and progression of tumors. However, since cancer is a progressive disease that results from accumulation of multiple mutations likely acting in concert, individual markers can only provide limited insights into cellular mechanisms that underlie tumorigenesis. For this reason, recent studies focus on identification of “sub-network markers”, that is, functionally associated genes that exhibit coordinate changes in molecular expression during cancer progression. However, expression of genes is most frequently interrogated at the mRNA level, which captures functional activity of genes only to a limited extent. Screening of protein expression, on the other hand, provides information on the abundance of functional gene products, but its scale is often limited compared to screening of mRNA expression. In this article, we develop a proteomics-driven computational method that searches for sub-network markers in human colorectal cancer, based on a seed of differentially expressed proteins identified by proteomic screening. Our results show that significant changes in the expression of these proteins is likely to be associated with coordinate changes in the expression of the genes whose products are functionally associated with these proteins. This analysis leads to novel insights in the synergistic processes that underlie tumorigenesis.

## Introduction

Colorectal cancer (CRC) is the second leading cause of cancer death in adult Americans [1]. Interest in this complex disease is represented by a very mature body of research, much of it at the genomic level. Yet the identification and verification of proteins that have a functional role in the patho-physiology of CRC remains an important goal as proteins directly mediate the functions dysregulated in the disease. Modern, high-throughput proteomic methods provide one way of profiling the significant changes in protein expression of tumor samples with respect to control, using tissue biopsies obtained from patients diagnosed with this disease [2]–[5].

Proteomic screening techniques are particularly useful for furthering the understanding of the mechanisms that underlie complex phenotypes like CRC, in that they provide information at the post-translational level. However, due to various biological and experimental constraints (*e.g.*, ascertainment bias and physical properties of proteins), proteomic methods may screen only a limited fraction of proteins and protein isoforms present in cells and tissues. We propose that this limitation may be mitigated through the integration of proteomic data with genome scale data sources, such as measurements of gene expression. In addition, protein-protein interaction (PPI) databases, which are rapidly growing in terms of both the quality and quantity of their annotations, provide another source of genome scale data integration [6]. Such integrative approaches can potentially lead to functional inference at the systems level, through identification of pathways and molecular sub-networks that are implicated in CRC.

In support of this approach, a recent review by Ideker and Sharan [7] summarizes studies that indicate that genes with a role in cancer tend to cluster together on well-connected sub-networks of protein-protein interactions. This suggests a hypothesis that the synergistic expression of multiple cancer-related genes at the level of mRNA can co-regulate the expression of proteins in their immediate “network neighborhood”. These differentially expressed proteins may be captured by expression proteomics experiments, thus their network neighborhood should provide an ideal starting place to search for sub-networks with a possible role in the disease.

The effectiveness of network-based approaches to the identification of multiple disease markers has been demonstrated in the context of various diseases, including Huntington's disease [8], the inflammatory response [9], and human breast cancer [10]. Furthermore, it was recently shown that “differentially expressed sub-network markers” were more accurate predictors of metastasis in breast cancer (compared to single gene markers) [11]. However, existing approaches are generally limited to mRNA expression data in terms of quantification of molecular expression, which captures post-transcriptional activity only to a limited extent [12],[13]. Consequently, inclusion of protein expression data in the search for sub-network markers has the potential to improve the effectiveness of systems biology approaches [14]. However, it remains largely unknown how a network-based approach may be enhanced when starting with proteomic data.

**Figure 1:**
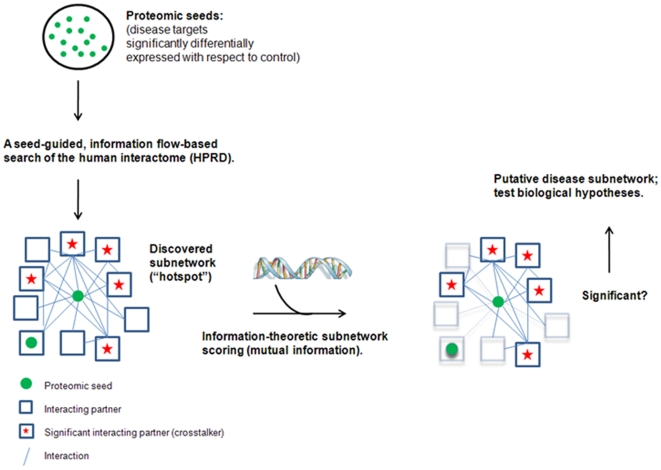
Schematic of an integrated, proteomics-first approach for the discovery of functional, candidate sub-networks in a disease phenotype. Disease targets significant for a phenotype (e.g. cancer) are used to seed an information-flow based search of the human interactome for candidate sub-networks subsequently classified as *crosstalkers* or *interactors*. Candidate sub-networks are then scored between test and control (e.g. normal vs. tumor) using the mutual information of aggregate mRNA expression data as a proxy for synergistic dysregulation. High-scoring sub-networks may be experimentally validated for their role in disease.

In this paper, we propose a novel computational approach that takes into account certain topological features of the interactome, namely connectivity and proximity, for searching the neighborhoods of proteomic targets to find significant sub-networks implicated in CRC. In doing so, we partly overcome (i) the bias inherent in proteomic profiling experiments, particularly those that are gel-based, which are typically limited to capturing changes only in relatively abundant proteins and (ii) the noise, missing data, and ascertainment bias in PPI data. This is accomplished by assessing the functional association between proteins based on the quantification of the statistical significance of *network crosstalk* through information-flow based modeling of the PPI network and development of a reference model that takes into account the network connectivity of proteomic targets. We hypothesize that identification of candidate sub-networks with a significant association to proteomic targets can reveal proteins that are not detected to be differentially expressed at the level of the proteome, but whose activity in the network may play a key role in maintaining the phenotype. Consequently, the proposed framework provides a means for expanding proteome expression data to infer a role for proteins that exhibit significant crosstalk to the proteomic targets. The flow of the proposed computational framework is illustrated in Figure 1.

A key objective of this study is to systematically elaborate a proteomics-driven approach as a sound method for inferring small sub-networks implicated in complex phenotypes, and ultimately make these methods practically available to a wider community of researchers working in this area. For this purpose, we ground our approach on the hypothesis that the observed fold change of the proteomic targets may be associated with the synergistic dysregulation of their interacting partners at the level of mRNA. From a computational perspective, our hypothesis is based on the premise that sub-networks which exhibit significant association with the proteomic targets should also show a significant change in activity between control and cancer. To test this hypothesis, we first score each protein in the network based on their crosstalk with the proteomic targets. In order to account for noise, incompleteness of data, and ascertainment bias, we also develop novel methods for assessing the significance of these “crosstalk scores”. Then, for each proteomic target, we identify a candidate sub-network that is composed of its interacting partners with significant crosstalk scores. Subsequently, using an information theoretic measure, we evaluate the synergistic differential expression of these candidate sub-networks between control and disease, based on changes in mRNA expression obtained from microarray experiments performed on tissue biopsies collected from a cohort of patients with CRC. Finally, using the sub-networks that exhibit significant synergistic dysregulation as features, we develop classifiers to predict disease class across different data sets.

The proposed computational approach for assessing functional association between proteomic targets and other proteins uses a random-walk based algorithm. Recently, Kohler *et al.* [15] and Chen *et al.* [16] used similar network algorithms to prioritize candidate disease genes implicated by linkage analysis in a variety of human diseases. Vanunu and Sharan [17] developed a global, propagation-based method that exploits information on known causal disease genes and PPI confidence scores. Their method more accurately recovered known disease gene relationships compared to several other extant methods. In contrast to these applications and rather than using raw scores obtained by such information flow based algorithms, we develop reference models to assess the statistical significance of these scores, with a view to identifying proteins that are *significantly* associated with proteomic targets. Furthermore, our biological hypothesis, which drives our approach, is that targets (proteomic or genomic) significant for the CRC phenotype may reside in or near cancer hotspots in the network, and thus present an ideal starting place to search for high-value sub-networks associated with the disease. Therefore, our computational approach does not rely on canonical disease-related genes or proteins; rather, it is a global, unbiased search that tries to identify network interactions statistically significant with respect to *all* targets in an experimentally-derived set.

Our previous work in this area [5] was limited in scope due to the lack of access to the topology of the commercial PPI we employed. This prevented us from assessing the importance of topology for sub-network generation, which is the primary focus of our computational approach in this study. Likewise, our network scoring and statistical hypothesis testing were all greatly limited in the previous work due to incomplete access to an unpublished microarray data. For the same reason we were practically prevented from iteratively adjusting network search parameters in the commercial software that would have generated a large list of candidate sub-networks for scoring.

Here we describe a new network search method for finding high-value candidate sub-networks associated with CRC. To overcome the limitations of the previous study and to permit independent evaluation of our methods, we utilize a public PPI (HPRD) and public microarrays (Gene Expression Omnibus) to evaluate performance using two independent sets of proteomic targets obtained by 2D-PAGE that are also publically available. We compare this result to that obtained using a set of CRC driver gene mutants as seeds for the network search. The basis for this test is the hypothesis that if mutated gene products map to cancer hotspots on the network, they would be similarly useful as seeds for our network search algorithm. To reveal the practical utility of our integrative approach, and to extend it beyond merely a theoretical computational framework, we validate by western blot several targets in a sub-network predicted by our method to be dysregulated, using a cohort of tissue biopsies not used in the original proteomic screen. Finally, we employ a cross-validation approach to compare the disease classification performance of the proteomic-versus genomic-derived sub-networks.

Our results show that the proposed proteomics-driven approach, as it integrates a variety of biologically relevant data, can identify significant sub-networks implicated in a complex phenotype, i.e. CRC. The definition of terminology frequently used in this paper is provided in Table 1.

**Table 1:** Definition of terminology used frequently in this paper.

Term	Definition
Proteomic seed	A protein that is significantly differentially expressed between tumor and control, as identified by proteomic screening.
Proteomic seed set	A set of proteomic seeds that are identified together in one proteomic screening cohort.
Network crosstalk	The degree of network proximity and connectivity between (groups) of proteins, modeled as the amount of “information flow” between these proteins in a PPI network.
Crosstalker	A protein that exhibits statistically significant network crosstalk with proteins in a particular proteomic seed set.
Interactor sub-network	A sub-network of the PPI network induced by the interacting partners of a particular proteomic seed.
Crosstalker sub-network	A sub-network of the PPI network induced by the interacting partners of a particular proteomic seed, which are also identified as crosstalkers with respect to the corresponding proteomic seed set.
Synergy or Synergistic dysregulation	Coordinate mRNA-level differential expression of a group of genes in the phenotype.

## Results

We searched the PPI network obtained from the Human Protein Reference Database (HPRD) for CRC-implicated sub-networks using two distinct sets of proteomic targets from Nibbe *et al.* [5] (n = 67) and Friedman *et al.* [2] (n = 55). Both sets contain significant targets of CRC obtained by a proteomic screen using tissue biopsies (tumor and matched controls) obtained from twelve and six patients, respectively (see Proteomic Methods for details of the screen performed in our lab). We call these targets *proteomic seeds*. The HPRD PPI network was downloaded from the HPRD website on September 2008 and contained 35023 binary interactions between 9299 proteins, as well as 1060 protein complexes consisting of 2146 proteins. We integrated the binary interactions and protein complexes using a matrix model (e.g., each complex is represented as a clique between the proteins in the complex), to obtain a PPI network composed of 42781 binary interactions among 9442 proteins. 60 of the proteomic seeds from the data of Nibbe *et al.* had at least one interaction in HPRD, while 37 of the seeds from the data of Friedman *et al.* had at least one interaction in HPRD. 14 of the proteins in the two seed sets were common.

For every protein in HPRD, our procedure assigns a score based on the protein's proximity and connectivity to all the seeds (see Materials and Methods). If the score is not significant (p<0.001) but the protein directly interacts with one or more of the seeds, we call it an *interactor*, whereas a *crosstalker* is any protein whose score is significant. Note that a crosstalker is generally (but not necessarily always) an interactor since a significant crosstalk score for a protein indicates that it is in the network neighborhood of one or more of the seeds, however, there are many interactors that do not qualify as crosstalkers. Overall, this procedure revealed 233 crosstalkers for Nibbe seeds, and 210 crosstalkers for Friedman seeds.

Subsequently, for each proteomic seed in each set, a candidate sub-network consisting of its interactors, termed the *interactor sub-network*, was obtained, resulting in a total of 55 interactor sub-networks (46 for Nibbe seeds exclusively, 23 for Friedman seeds exclusively, and 14 additional sub-networks for both). Similarly, for each seed in both sets, a *crosstalker sub-network* was obtained. Thus, for every seed there are two corresponding sub-networks, an interactor sub-network and a crosstalker sub-network. The proteins in an interactor sub-network are merely characterized by their direct interactions with the corresponding proteomic seed. By contrast, proteins in a crosstalker sub-network are characterized by their degree of functional association with *all* proteomic seeds.

### Relationship of Expression between Crosstalkers and Individual Proteins in HPRD at the Level of mRNA

We evaluated the individual differential gene expression of each crosstalker identified using the Nibbe and Friedman proteomic seeds using two microarray datasets obtained from GEO (GSE10950 & GSE8671). GSE8671 represents 64 experiments using mRNA isolated from tissue biopsies obtained from 32 patients (matched tumor and adjacent normal mucosa) performed on an Affymetrix GeneChip (Human U133 Plus 2.0). Similarly, GSE10950 represents 48 experiments on matched tissue biopsies (24 patients) performed on an Illumina array (Human ref-8, v2.0).

**Figure 2:**
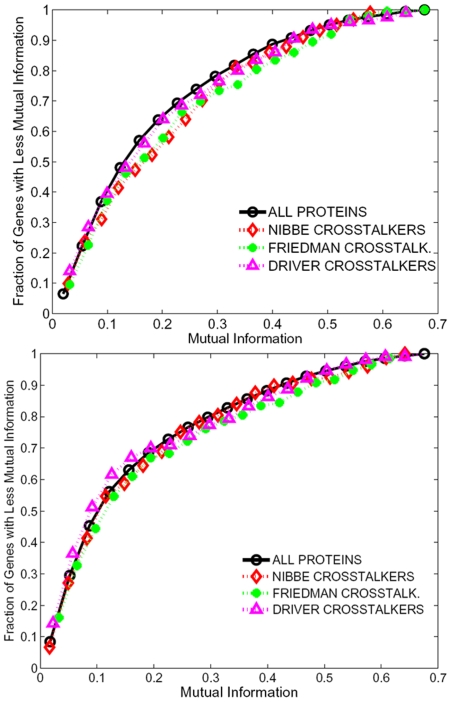
Crosstalkers are not significant at level of *individual* mRNA expression. Cumulative distribution of differential expression for crosstalkers identified using two proteomic seeds (Nibbe *et al.*, Friedman *et al.*), a seed of CRC driver genes (Sjöblom *et al.*), and all proteins in the HPRD PPI network, as quantified by mutual information with phenotype, using GSE8671 and GSE10950.

The cumulative distribution of individual differential expression scores for proteomic seeds, (and a seed of CRC driver genes discussed later), as well as all proteins in the network computed as described in the Materials and Methods section, is shown in Figure 2 (please see the Materials and Methods section for details on how differential expression is quantified). As seen in the figure, we found no significant difference in the distribution of individual differential expression of the crosstalkers, as compared to the distribution of differential expression of all proteins in the HPRD network. This observation indicates that at the level of *individual* genes, significant network crosstalk with proteomic seeds in CRC is not associated with transcriptomic dysregulation in CRC.

### Synergistic Regulation of Sub-Networks Induced by Proteomic Seeds

**Figure 3:**
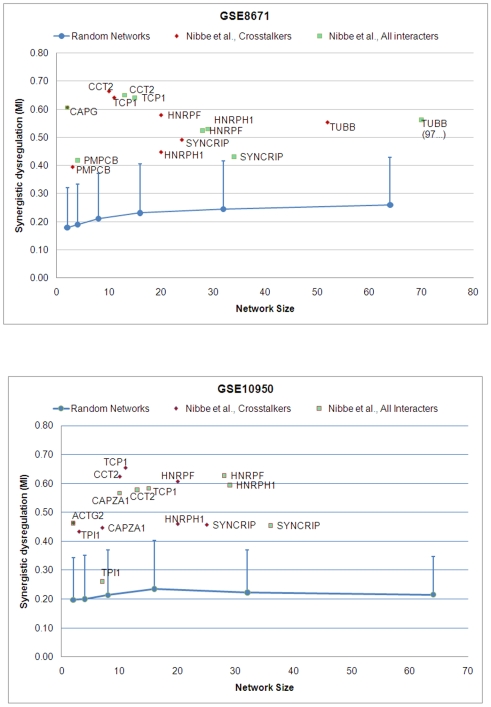
Synergistic dysregulation versus network size for candidate sub-networks associated with proteomic seeds obtained from Nibbe *et al*. Sub-network dysregulation (i.e. mutual information of sub-network mRNA expression profile with phenotype class) versus network size for candidate sub-networks. All interactors (green squares) and crosstalkers (red diamonds) were scored using (a) GSE10950 and (b) GSE8671. The blue lines represent the linear interpolation of the means of the estimated null distributions computed for random candidate sub-networks of size 2,4,8,16,32, and 64, using the respective arrays (see Materials and Methods for details). Vertical bars represent one standard deviation from the mean.

For the purpose of discussion we will refer to a sub-network by the proteomic seed that induced the sub-network (e.g. *TCP1*). For each version of each sub-network we computed the mutual information (MI) of each sub-network between control and tumor using the mRNA expression data from microarrays GSE10950 and GSE8671 (see Computational Methods), and we used this score to estimate the significance of the various networks in differentiating the phenotype (Figure 1). The comparison of mutual information for the two versions of each sub-network associated with the Nibbe seed is shown in Figure 3. We plotted the results only for those (crosstalker) sub-networks where the mutual information exceeded 0.35 (approximately 1σ from random mean). The purpose of this analysis is to understand how the synergy of each crosstalker sub-network compares to that of its corresponding interactor sub-network. The MI and significance scores for all sub-networks can be found in Supplemental Table S1.

Of the 46 candidate sub-networks associated with Nibbe proteomic seeds, 10 unique interactor sub-networks (green squares) exhibited significant MI scores. For five of these sub-networks (*CCT2*, *TCP1*, *SYNCRIP*, *HNRPF* and *HNRPH1*) the crosstalker version of the sub-networks was found to have enhanced MI on one or the other microarray datasets. Two crosstalker sub-networks (red diamonds), *CCT2* and *TCP1*, show improvement over their corresponding interactor sub-network on both arrays. Notably, on GSE10950, the mutual information score of the *TPI1* crosstalker sub-network is significant, while the corresponding interactor sub-network failed to show significance.

**Figure 4:**
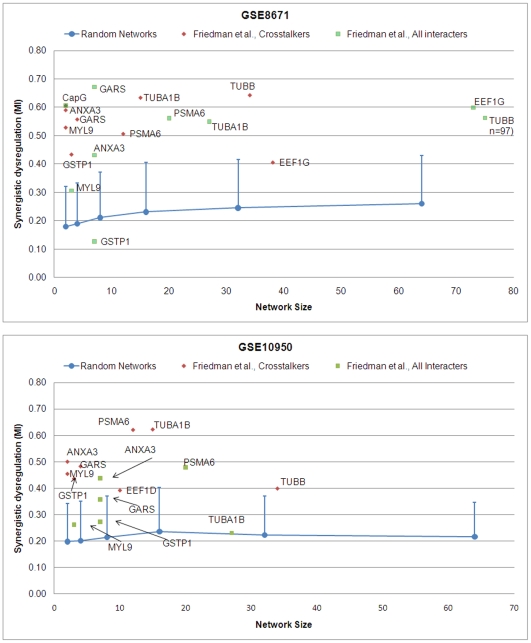
Synergistic dysregulation versus network size for candidate sub-networks associated with proteomic seeds obtained from Friedman *et al*. Please see Figure. 3 for annotation.

Figure 4 shows the corresponding plots for the Friedman proteomic seeds. Here, seven unique interactor sub-networks have significant MI scores; two of them (*ANXA3* and *PSMA6*) were common to both sets of microarray data. For the Friedman seeds, the crosstalkers for candidate sub-network *TUBA1B* showed dramatically increased mutual information compared to its interactor network. Furthermore, four other crosstalker sub-networks (associated with *MYL9*, *GARS*, *ANXA3* and *GSTP1*) all revealed much higher synergy compared to their corresponding interactor sub-networks, two of which (*MYL9*, *GSTP1*) failed to show significance on either array. We discuss a possible explanation for these findings in the Discussion section.

**Figure 5:**
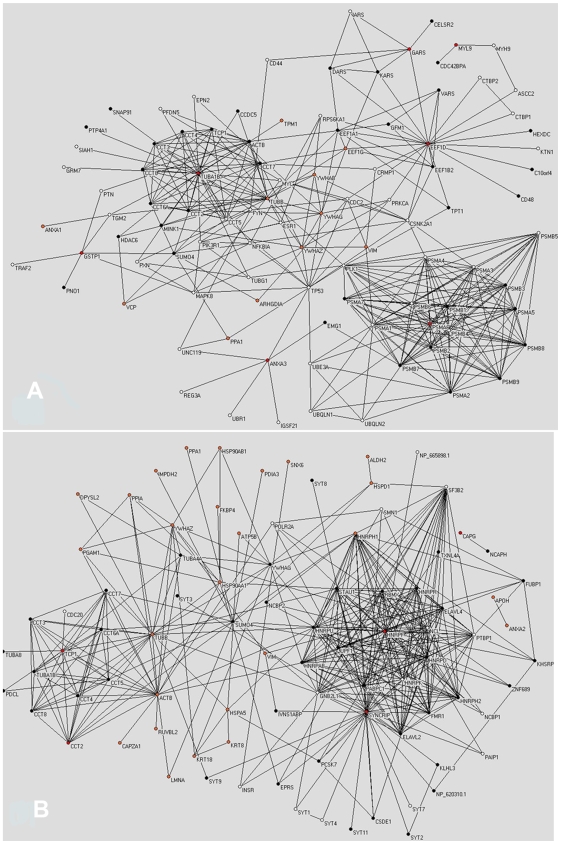
Significant sub-networks induced by proteomic seeds. Network graph visualization of sub-networks induced by Friedman seed, scored using GSE10950 (a) and Nibbe seed, scored using GSE8671 (b). Proteomic seeds that induced a significant crosstalker sub-network are shown in red, other proteomic seeds are shown in orange, *crosstalkers* are black and *interactors* are white. Visualization was performed with the Pajek software.

Figures 5a and 5b show unions of crosstalker sub-networks associated with the Friedman and Nibbe seeds, respectively, for which the synergy was higher than the corresponding interactor sub-network. The graphs reveal that many proteomic seeds reside within or near dense sub-networks of crosstalkers.

### Post-Trancriptional Dysregulation of TCP1 Sub-Network

We observed that several of the sub-networks generated using the two proteomic seed sets contained proteins in common. In particular, certain sub-units of the *TCP1* complex exhibited marked crosstalk in the sub-network induced by *CCT2* in the Nibbe seed, and *TUBA1B* in the Friedman seed (Figure 4). In addition, we had previously shown [5] that certain sub-units of this complex (*CCT3*, *CCT5*, and *CCT7*) were also significant for the late-stage CRC phenotype, as revealed by a similar network scoring methodology but using a commercial PPI unrelated to HPRD.

**Figure 6:**
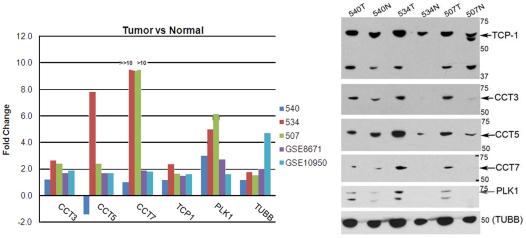
Validation of select targets predicted to be dysregulated in *TCP1* sub-network. Immunoblot data were obtained from three (540, 534, 507) late-stage matched (N = normal/T = tumor) patient tissue biopsies not used in the original proteomic screen by Nibbe *et. al.* Values are in kilodalton (kDa). GSE8671 and GSE10950 represent the ratio of the mean mRNA value (tumor/normal) from the respective microarray array. Fold change was determined by densitometry.

*TCP1* (or *TCPα*) is a hetero-oligomeric complex comprised of two stacked ring structures, each composed of eight known subunits and plays a functional role in maintaining the CRC phenotype. Specifically, it was shown [18] to be required for the proper biogenesis of *PLK1*, a kinase that has a critical role in cytokinesis. However, other than their role as sub-units in the formation of the TCP complex little is known about the independent role, if any, of these sub-units in CRC [19]. Consequently, these targets present an opportunity for follow-on mechanistic studies. For this reason, we verified the protein expression of *TCP1*, *CCT3*, *CCT5*, *CCT7*, and *PLK1* by western blot in a separate cohort of three patient sample pairs not used in screening phase, and compared this to the average expression at the level of mRNA (Figure 6). Consistent with our hypothesis, the data indicate co-regulation at the level of mRNA and protein, but also reveal the wide variability of expression of these targets among individual patients. *CCT3* and *CCT7* were dramatically over-expressed in two patients (507 and 534), but less so in patient 540, which was similar to the pattern for *PLK1*.

### Synergistic Dysregulation of Sub-Networks Induced by CRC Driver-Gene Seeds

Although these data show that proteomic seeds are well-suited for identifying synergistically dysregulated sub-networks, we wished to investigate the power of genetically identified seed sets in discovering significant sub-networks. As CRC is commonly thought to be caused by the accumulation of somatic mutations, a number of cancer research labs have collaborated to conduct whole genome sequencing to identify the genes thought to be “drivers” in cancer, i.e. those represented by the set of genes that appeared most frequently mutated in a robust cohort of clinical biopsies. The results of one such study on human breast and colon cancer were recently reported by Sjöblom *et al.* [20]. We hypothesized that the gene products of the CRC driver genes reported in this study would be located at hotspots in the interactome. Further, if the mutations lead to dysregulation of neighboring genes at the level of mRNA, then the seed should reveal significant sub-networks using our method. Additionally, since there is less bias in PCR sequencing and high genome coverage, at least as compared to proteomic profiling, we supposed that driver gene seeds (n = 42) might be superior both in terms of the number and significance of the sub-networks identified.

**Figure 7:**
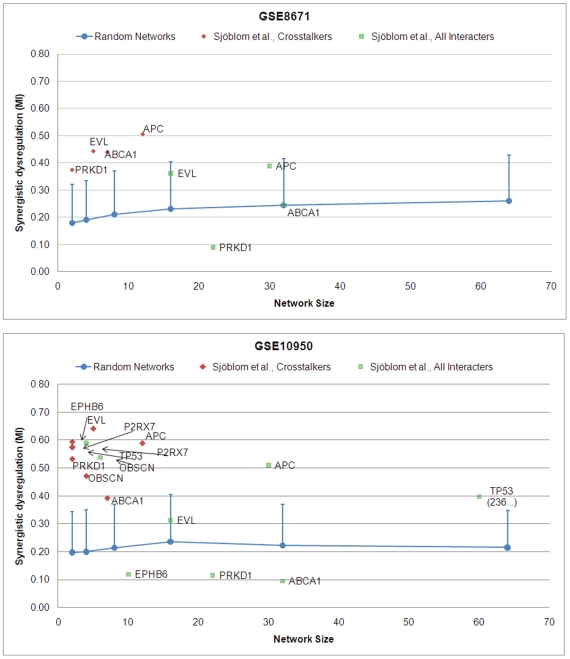
Synergistic dysregulation versus network size for candidate sub-networks associated with the CRC driver gene seeds obtained from Sjöblom *et al*. Please see Figure 3 for annotation.

As shown in Figure 7, when scored by GSE8671, only four significant sub-networks were found. Strikingly, for every one of them, only the crosstalker sub-networks were significant. Using GSE10950, seven sub-networks of crosstalkers were significant, including all four found on GSE8671. For all but two of the sub-networks (*P2RX7*, *OBSCN*), the crosstalkers show substantially higher synergistic differential expression as compared to their interactor counterparts. Notably, *APC*, a tumor suppressor gene widely viewed as the “gate-keeper” in CRC, was associated with a significantly dysregulated sub-network with respect to both arrays, and of all the genes in the driver seed it was found to be mutated in the highest percentage (90%) of the clinical samples. This expected finding may be viewed as a positive control for our analytical method.

In terms of the overall number of significant sub-networks identified, however, there was no apparent improvement using the driver gene seed set versus either proteomic seed set. Additionally, a number of the significant crosstalk sub-networks identified by the proteomic seeds show markedly higher synergy (MI>0.60) than all but one (*EVL*) of the sub-networks found by the driver gene seed.

### Classification Performance of Sub-Networks as Features

**Figure 8:**
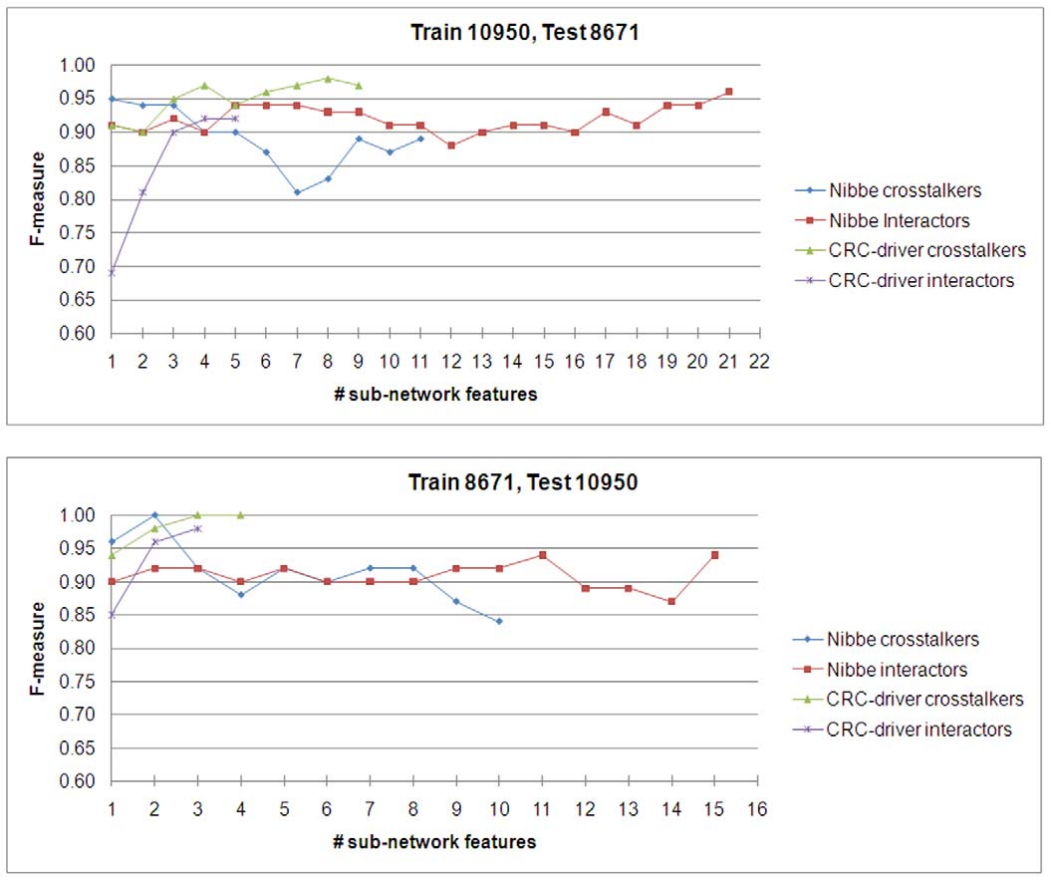
Cross-validation performance comparison of sub-network based classifiers. The sub-networks induced by proteomic and genomic seeds were first ranked by mutual information with phenotype (MI). Then the normalized mRNA expression values for the genes were aggregated to compute a feature for each sub-network with significant MI. These features were used to train an SVM-based classifier to distinguish normal from tumor using GSE10950, and then cross-validated on GSE8671 (a), and vice-versa (b).

We evaluated the quality of the crosstalker versus interactor sub-networks in terms of their ability to classify tumor versus control on the microarrays, using an SVM-based classifier in a cross-validation approach (see Materials and Methods). The significant sub-networks in each group were first ranked by MI, and the features were valued by superposing the mRNA expression values of each gene in the sub-network. When trained on GSE10950 and validated on GSE8671, proteomic crosstalkers outperformed the interactor sub-networks (both proteomic and genomic) when the number of features used to train the classifier was three or less. Beyond three features, both the proteomic interactor and CAN (candidate CRC driver genes) crosstalker sub-networks outperformed the proteomic crosstalkers (Figure 8a). Performance was similar when the training and validation sets were reversed, although the performance of proteomic crosstalkers dropped when more than two sub-networks were used for classification (Figure 8b). The raw classification data are provided in Supplemental Table S1.

## Discussion

We have shown that proteomic targets showing significant expression changes for a complex phenotype, such as CRC, provide valuable inputs for our algorithms designed to discover phenotypically significant sub-networks with connectivity and proximity to these targets. In addition, certain crosstalker sub-networks, when scored with respect to phenotype by the measure of mutual information, display significant differential synergistic expression at the level of mRNA with respect to the seed targets. When these implicated sub-networks contain proteins with no known role in the disease, they present new opportunities for follow-on mechanistic experiments to verify the *in silico* inference of biological significance in the disease. This point cannot be over-emphasized, because in our view the promotion of a *candidate*, disease-associated sub-network to an functional sub-network with a validated role in disease must be accomplished by wet lab experiments.

As mentioned in the previous section, with respect to the proteomic seeds, a number of the same sub-networks showed significance (>1σ from background) when scored by either GSE10950 or GSE8671. With respect to the driver gene seed, every sub-network that showed significance when scored by the GSE8671 array was also found to be significant when scored by the GSE10950 array. One explanation for why the sub-networks with respect to a given set of proteomic seeds did not show complete redundancy between arrays is that the microarrays represent experiments performed on different pathologic stages of CRC tumors, very early stage in the case of GSE8671 (adenoma) versus a more established tumor in GSE10950 (primary). The pathologic stage of the proteomic samples in the Nibbe seed was homogenous late stage CRC (Duke's D) while the Friedman seed was a mix of mid to late stage samples (Duke's B–D). This highlights a potential limitation of an integrated –omics approach, namely, it is often difficult to establish an optimal match of the biology underlying the measures made at the level of the proteome and transcriptome. However, in our case, if the sub-networks become dysregulated early in the disease and have a role in maintaining the phenotype through later stages, this limitation can turn into an opportunity for development of hypotheses regarding the mechanisms of the progression of CRC. In particular, the complete overlap of crosstalk sub-networks between arrays observed with the driver gene seed indicates the synergistic activity of these sub-networks may be independent of pathologic stage.

We also noted that only a relatively small fraction of the seeds induced significant sub-networks, either interactors or crosstalkers, and this was the case for both the proteomic and the genomic seeds. One potential explanation for this observation is that current human PPI networks capture only a very small fraction of all protein relationships in the human interactome [21], and therefore cannot be expected to reveal a significant sub-network for every experimentally determined seed. As these networks improve, we expect their value in uncovering interesting biology will only grow.

The classification performance indicates that experimentally-derived proteomic disease targets combined with our network search algorithm can discover high-valued sub-networks for mechanistic *in vivo* verification. This was consistent with our hypothesis, and supports the claim that a proteomic seed can identify sub-networks that provide additional pathways of interest (e.g CCT2, TCP1). To strengthen this claim, in an independent cohort of patient biopsies, we validated the differential expression of several targets in the TCP-1 sub-network, predicted by our model to be coordinately dysregulated.

The genomic seed showed excellent classification performance, and crosstalkers were superior in most instances to their corresponding interactor sub-networks, consistent with our computational hypotheses. When three or more features were used to train the classifier they were also better than the proteomic crosstalkers. However, this result is not entirely unexpected as the proteomic data has low coverage and may lack key seeds and thus may lack important sub-networks. However, the favorable classification performance of the genomic-derived sub-networks may be viewed as a positive control for this experimental approach. Alternatively, it is unlikely that all relevant sub-networks are regulated at the level of transcription, and this may reduce the number of significant sub-networks discoverable by our approach. Never-the-less, the approach can be generalized to many proteomics expression data sets to discover novel sub-networks dysregulated in many complex diseases.

In many classification applications, high dimensionality is an important problem and it is often desirable to be able to choose a small number of features that will provide reasonable performance (to overcome “curse of dimensionality”). In this respect, the classification performance provided by only a few sub-networks is indeed very promising, in that “crosstalk to proteomic targets” may actually provide a shortcut to the identification of a compact set of useful sub-network features. As our classification experiments were carried out in a cross-classification setting, the high accuracy of classification using up to three sub-networks indicates that the most significant crosstalker sub-networks were highly reproducible. Reproducibility is an important concern in classification applications, since if the sub-network features that are used are not reproducible across datasets, this will result in over-fitting. In this regard, the use of proteomic data can also be considered a tool for obtaining useful biological insights for feature selection.

## Materials and Methods

### Proteomic Methods

#### Target screen

The Nibbe et al. proteomic targets were determined using two gel-based screens of twelve and six, respectively, late-stage CRC tumor tissue biopsies (with matched adjacent normals) obtained from the Case Comprehensive Cancer Center. Briefly, the biologically significant spots between normal and tumor were identified by image analysis of the 2D-gels. The spots were then robotically excised, digested by trypsin, and the peptide sequences determined by LC-MS/MS. Parent proteins were subsequently identified by database search. Full experimental details as well as the lists of the targets identified in both screens can be found at Nibbe *et al* [5]. It merits emphasis that the targets selected for network analysis were highly significant given the stringent p-values used (<0.01) at the level of peptide and protein identification. The targets in the Friedman seed were similarly identified (see the Methods section of Friedman *et al.*) on a smaller cohort of paired biopsies (n = 6) of mixed stage CRC.

#### Western blot

The tissue samples were thawed and homogenized in Lameali buffer with a Polytron mixer. Protein concentration was determined by a kit (Amersham Biosciences, 2D-Quant). Aliquots were diluted to 5 ug/ul,and stored at −80°C. 15 ug of total protein was separated by 1D-PAGE on homogeneous 10% gels. The protein was immediately transferred to a nitrocellulose membrane (40 mA for 4 hours on ice). Membranes were blocked overnight at 4°C with 5% milk in TBS-T, washed 2× with TBS-T at room temperature, and subsequently incubated with the primary antibody (Sigma) overnight at 4°C. The membranes were once again washed 2× at room temperature and incubated with the secondary antibody (Cell Signaling) for two hours. The membranes were then washed 3× with TBS-T, incubated with ECL reagent (Pierce) and exposed from one to ten minutes (protein dependent). Fold change was determined using the 2D-QUANT software (Amersham Bioscience).

### Computational Methods

The computational framework for integrating proteomic, transcriptomic, and interactomic data to discover sub-networks implicated in complex phenotypes is shown in Figure 1. As seen in the figure, we first identify disease targets with significant differential expression with respect to control, via proteomic screening as described above. Once these targets, called proteomic seeds, are identified, we map these seeds on the PPI network obtained from HPRD to identify proteins that are functionally associated with the proteomic seeds.

In order to develop biologically sound measures to quantify the functional association between proteins, we develop information flow based algorithms to compute *crosstalk* scores, which capture network proximity and connectivity to proteomic seeds. We discuss this procedure in Subsections A and B. In order to account for experimental artifacts, incompleteness of data, and ascertainment bias, we use Monte Carlo simulations to assess the significance of the crosstalk scores computed by these algorithms. Our statistical evaluation scheme is based on a reference model that captures the basic characteristics of the proteomic seeds, in terms of the number of seeds and their degree distribution. This procedure is described in Subsection C.

Subsequently, for each proteomic seed, we construct two “candidate sub-networks”: (i) sub-network induced by all interacting partners of the seed protein, (ii) sub-network induced by the interacting partners that have significant crosstalk scores (in our experiments, we use a *p*-value cut-off of 0.001 to determine “significant crosstalkers”). Finally, we evaluate the mutual information score of each candidate sub-network with respect to the phenotype of interest (in this paper, CRC), using mRNA expression data for test and control samples. For this purpose, we use an established information-theoretic scheme that quantifies synergistic differential expression in terms of the mutual information between the aggregate expression of the sub-network and disease classes across samples. This procedure is explained in Subsection D. In order to assess the statistical significance of synergistic differential expression, we also use Monte Carlo simulations based on reference models that accurately capture the basic topological characteristics of each sub-network. This procedure is explained in Subsection E. We then use identified sub-networks to develop classifiers for predicting disease class in CRC. This procedure is explained in Subsection F.

### Relationship between Synergistic Expression, Functional Association, and Network Topology

Systematic studies of differentially expressed genes in certain phenotype classes show that these genes are related to each other in molecular networks, composed of protein-protein interactions, transcriptional regulatory interactions, and metabolic interactions [22]. In one of the early algorithmic studies, Ideker *et al.* [23] develop a method for identifying differentially expressed metabolic sub-networks with respect to GAL80 deletion in yeast. This method is based on searching for connected groups of enzymes within the yeast metabolic network, such that the aggregate differential expression of genes coding these enzymes is statistically significant. Variations of this method prove useful in identifying multiple gene markers implicated in a variety of diseases, including prostate cancer [24], melanoma [25], and diabetes [26]. Building on these results, information theoretic schemes for assessing synergistic differential expression are also shown to be effective in network based disease classification [11],[27].

While differential network analysis is effective in identifying multiple gene markers, most of the existing methods utilize network information to primarily find the genes that are connected, hence potentially related to each other. In other words, these approaches do not take into account network topology, connectivity patterns, or degree of connectivity between proteins. This is because (i) much of the available network information is noisy and incomplete [28], therefore, connectivity patterns cannot be interpreted as well-defined wiring schemes, and (ii) network models (particularly, high-throughput protein-protein interactions) provide only a high-level qualitative description of the information flow in the cell. However, several studies show that variations in molecular expression can be interpreted in terms of network topology (*e.g*, subunits of a protein complex are co-expressed significantly over a time course [29], functional similarity of proteins correlates with proximity in a network of interactions [30],[31].

Motivated by these considerations, we develop network-based scoring schemes to quantify the crosstalk between proteomic seeds and the rest of the proteins in a network of interactions. Based on the premise that synergistic changes in transcriptional expression may be associated with significant changes in proteomic activity, we expect that proteins that demonstrate significant crosstalk with proteomic seeds will be good candidates for being implicated in the phenotype of interest. In order to assess the crosstalk between a group of proteomic targets and any other protein in the network accurately, we develop information flow based algorithms, as discussed in the next section.

### Network Crosstalk: Capturing Functional Association via Connectivity and Proximity

Let *G = (V,E)* be a network of protein interactions, where *V* consists of the proteins in the network, and an undirected edge *uv*∈*E* represents an interaction between proteins *u*∈*V* and *v*∈*V*. For convenience, we also define *N*(*v*) as the set of interacting partners of protein *v*∈*V*, *i.e.*, *N*(*v*) = {*u*∈*V*: *uv*∈*E*}. Let *S*⊆*V* be the set of proteomic seeds, *i.e.*, the proteins that are identified by proteomic studies to exhibit significant fold change with respect to the phenotype of interest. Our objective is to compute a score *α*(*v*) for each protein *v*∈*V*, to quantify the network crosstalk between *v* and the proteins in *S*. Here, network crosstalk is used as an indicator of functional association between proteins.

In order to develop a biologically sound measure of network crosstalk, we rely on the following observations: (i) Functional similarity between two proteins, as measured by semantic similarity of Gene Ontology annotations [32], is significantly correlated with their network proximity, as measured by the shortest path (number of hops) between these proteins [30],[31]. (ii) Existence of multiple alternate paths between two proteins is an indicator of their functional association, since functional multiple paths are often conserved through evolution owing to their contribution to robustness against perturbations, as well as amplification of signals [33].

To incorporate both the number of hops and multiple alternate paths into the assessment of crosstalk between proteins, we use an information flow based algorithm based on random walks with restarts [34]. This algorithm can be considered a generalization of Google's well-known page-rank algorithm [35]. Furthermore, a special case of the proposed crosstalk score, when |S| = 1, is a network proximity measure [34] known to be closely related to commute distance and effective resistance [36] in graphs. Similar graph-theoretic measures are also used to identify functional modules in PPI networks [37], annotation of protein function [38], and prioritization of disease genes [15]–[17].

We assign crosstalk scores to all proteins in the network for a given *S* by simulating a random walk as follows. The random walk starts at a randomly chosen protein in *S*. At each step, when the random walk is at some protein *v*, it either moves to an interacting partner of *v* with probability 1−*r*, or it restarts at a protein in *S* with probability *r*. Here, the parameter 0≤*r*≤1 is called the restart probability (in our experiments, we use *r* = 0.5). For each move, the interacting partner to be moved to is selected uniformly at random from *N*(*v*). However, the move probabilities can also be adjusted to reflect the confidence of each interaction, so that more reliable interactions contribute more to the quantification of crosstalk. In other words, one can define the probability of a move from *v* to *u* as *P*(*u*,*v*) = *w*(*u*,*v*)/Σ*_u′∈_*_*N*__(*v*)_ *w*(*u′*,*v*) if *u*∈*N*(*v*), 0 otherwise. Here, *w*(*u*,*v*) denotes the reliability of the interaction between *u* and *v*. Similarly, for each restart, the protein to be restarted is selected uniformly at random from *S*. These probabilities can also be adjusted to reflect the significance of the fold change of each protein in *S*, so that proteins with more significant fold change are considered as more reliable seed proteins. In other words, one can define the probability of restart at *u*∈*V* as ρ(*u*) = z_P_(*u*)/Σ*_u′∈S_*z_P_(*u′*) if u∈*S* and 0 otherwise. Here, z_P_(*u*) denotes the z-score of the fold change of *u* with respect to the phenotype of interest, based on proteomic screening.

Based on this random walk model, we define the crosstalk between the proteins in *S* and each protein *v*∈*V* as the relative amount of time spent at *v* by such an infinite random walk, or equivalently, the probability that the random walk will be at protein *v* at a randomly chosen time step after the random walk proceeds for a sufficiently long time. More precisely, let *α*_t_ denote a |*V*|-dimensional vector, such that *α*_t_(*v*) is equal to the probability that the random walk will be at protein *v* at step *t*, where ∥*α*_t_∥_1_ = 1 (here, ∥.∥_1_ denotes the 1-norm of a vector, defined as the sum of magnitudes of its elements). Let *P* denote the stochastic matrix derived from network *G* = (*V*,*E)*, i.e., *P*(*u*,*v*) = 1/|*N*(*v*)| if *uv*∈*E*, 0 otherwise. Then, we have
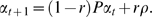
(1)where ρ denotes the restart vector with ρ(*u*) = 1/|*S*| for *u*∈*S*, and 0 otherwise. Then, letting α_0_ = ρ, the vector containing the crosstalk scores for each node in the network is given by α = lim*_t→∞_* α_t_. Observe that this formulation lends itself to an iterative algorithm to compute crosstalk scores efficiently, where each iteration requires *O*(|*E*|) time, since *P* is a sparse matrix with 2|*E*| non-zero entries.

Note that, when *r* = 0, α is equal to the eigenvector of *P* that corresponds to its largest eigenvalue (with numerical value 1), *i.e.*, α(*v*) is exactly equal to the page rank of *v* in *G* for all *v*∈*V*. Therefore, the crosstalk score of a protein is not only an indicator of its connectivity and proximity to seed proteins, but it is also influenced by the centrality of the protein in the network. In order to account for such sources of bias, as well as the choice of parameter *r* (in our experiments, we use *r* = 0.5), we adjust the crosstalk scores statistically as we discuss in the next section.

### Dealing with Experimental Artifacts, Ascertainment Bias, and Incomplete Data

Due to variability in physical properties of proteins and other experimental artifacts, it is likely that there will be significant ascertainment bias in the selection of proteomic seeds, as well as the availability of interaction data for each protein [39]. Indeed, our results show that the seed proteins extracted by proteomic screening are likely to be highly connected in the PPI network derived from HPRD. More specifically, the 60 proteins that are identified to have significant fold change (*p*<0.01) in late stages of human colorectal cancer have 24.1 interactions in HPRD on an average, while the average degree of a protein in the HPRD network is 9.1. Consequently, highly connected proteins in the network are likely to be assigned artificially high crosstalk scores just by chance. Since available network data is often incomplete and prone to ascertainment bias, these effects are likely to amplify the ascertainment bias and skew the results toward well-studied proteins. However, we are very interested in finding those proteins that are relatively less characterized but may provide novel insights into phenotype. Therefore, the crosstalk scores described above need to be assigned significance scores based on reliable statistical models.

In order to deal with such experimental and data-related sources of bias, we use a reference model that captures the degree distribution of seed proteins accurately. Namely, for a given seed set *S*, we generate a random instance *S* ^(i)^ representative of *S* as follows. For every protein *u*∈*S*, we create a bucket *B*(*u*) of proteins in the network, such that ∪*_u_*_∈*S*_ *B*(*u*) = *V* and *B*(*u*)∩*B*(*u*′) = ∅ for all *u*, *u* ^′^∈*S*. Here, protein *v*∈*V* is assigned to bucket *B*(*u*) if |*N*(*v*)−*N*(*u*)|≤|*N*(*v*)−*N*(*u′*)| for all *u′*∈*S* and ties are broken randomly. Then, we construct *S* ^(i)^ by choosing one protein from each bucket uniformly at random, so that |*S* ^(i)^| = |*S*|. Observe that each bucket consists of proteins that have similar number of interactions with a particular seed protein; therefore, each seed protein is represented in *S* ^(i)^ by exactly one protein in terms of its number of interactions. Consequently, the expected total degree of the proteins in *S* ^(i)^ is likely to be very close to the total degree of the proteins in *S*. Once a random instance *S* ^(i)^ is generated, we compute the corresponding crosstalk vector α^(i)^ by letting *ρ* ^(i)^(*u*) = 1/|*S* ^(i)^| for *u*∈*S* ^(i)^, and 0 otherwise.

Repeating this procedure *n* times, where *n* is sufficiently large (we use *n* = 1000 in our experiments), we obtain a sampling {α^(1)^, α^(2)^, …, α^(n)^} of the null distribution of crosstalk scores, with respect to seed sets that are representative of *S* in terms of their size and degree distribution. We then estimate the mean μ_S_ = Σ_1≤i≤n_α^(i)^/*n* and standard deviation σ_S_^2^ = Σ_1≤i≤n_(α^(i)^−μ_S_)^2^/(n−1) of the null distribution of crosstalk scores for *S* using this sample. Subsequently, we compute adjusted crosstalk scores
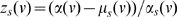
(2)for each protein *v*∈*V*. These adjusted crosstalk scores represent the statistical significance of the crosstalk between each protein and the proteins in the seed set, accounting for the centrality of the protein the network, as well as the degree distribution of seed proteins.

### Assessing Synergistic Dysregulation of Candidate Sub-Networks

Once all proteins in the network are scored according to their crosstalk with proteomic seeds, we construct candidate sub-networks as follows:

1. **Interactor sub-networks:** For each proteomic seed *u*, the sub-network induced by its interacting partners in the network (*N*(*u*)) is considered a candidate sub-network, based on the hypothesis that significant changes in the expression of a protein may be associated with synergistic changes in the transcriptional expression of proteins in its neighborhood.
2. **Crosstalker sub-networks:** For each proteomic seed *u*, the sub-network induced by the proteins in *N*(*u*) that have significant adjusted crosstalk scores with respect to *S* is considered a candidate sub-network, based on the hypothesis that sub-networks composed of proteins with significant crosstalk to the proteomic seeds (as opposed to solely interacting with one proteomic seed) are likely to exhibit significant synergistic differential expression.

Formally, the set of candidate sub-networks is defined as *C*(*S*) = {*N*(*u*):*u*∈*S*}∪{*N* ^*^(*u*):*u*∈*S*}, where *N* ^*^(*u*) = {*v*∈*N*(*u*): *z*_S_(*v*)>*z* ^*^}. Here, *z* ^*^ denotes the cut-off for adjusted crosstalk scores to be considered significant. In our experiments, we use *z* ^*^ = 3.45, to reflect a *p*-value cut-off of 0.001, under the assumption of normally distributed crosstalk scores.

For each candidate sub-network *Q* in *C*(*S*), we quantify the synergistic expression of the proteins in *Q* using an information-theoretic scheme developed by Chuang *et al.* [11]. Namely, for protein *v*∈*V*, let *e*(*v*) denote the properly normalized *m*-dimensional mRNA expression vector, provided by genome-scale transcriptomic screening of *m* disease and control samples. Let *c* denote an *m*-dimensional binary vector indicating the phenotype class of each sample, such that *c*(*i*) = 1 if the *i*th sample is diagnosed with the disease, 0 otherwise. Furthermore, define the aggregate expression vector *e*(*Q*) for the sub-network induced by set of proteins *Q* as
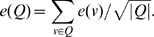
(3)


Then, the synergistic differential expression ϕ(*Q*) of the genes coding for proteins in *Q* with respect to the phenotype of interest is given by the mutual information between *e(Q)* and *c*, *i.e.*,
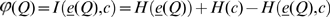
(4)Here, *e*(*Q*) denotes a discrete-valued vector obtained by quantizing *e*(*Q*) into *k* bins, *H(x*) denotes the entropy of a discrete-valued vector *x* over a finite alphabet *A*, *i.e.*, *H*(*x*) = Σ*_a∈A_*−*p*(*a*)log(*p*(*a*)), and *p*(*a*) = |{*i*:*x*(*i*) = *a*}|/*m* (in the context of our problem, *A* represents the set of bins). In this paper, we use *k* = 6, since this value of *k* was found to provide reasonable estimates for mutual information in our experiments.

### Statistical Significance of Synergistic Dysregulation

Finally, we assess the statistical significance of synergistic differential expression for each candidate sub-network. In order to do so, for a given *Q*∈*C*(*S*), we generate a null distribution for synergistic differential expression of sub-networks that reflect the topological properties of *Q*. Since *Q* is composed of proteins that are connected to each other via a single protein (that is, the corresponding proteomic seed), the null distribution should also be derived from sub-networks that consist of the same number of proteins in *Q*, which are connected to each other through a single protein in the network. Therefore, we first construct a bag *D* of proteins in the network with degree at least *|Q|*, *i.e*, *D* = {*v*∈*V*:|*N*(*v*)|≥|*Q*|}. Subsequently, we choose a protein *v* from *D* uniformly at random. Finally, we choose |*Q*| proteins uniformly at random from *N*(*v*) to construct a random instance *Q* ^(*i*)^ representative of *Q*. Repeating this procedure *n* times (in our experiments, we use *n* = 1000) and computing ϕ(*Q* ^(*i*)^), we obtain a null distribution of synergistic differential expression for sub-networks similar to *Q*. Observe that, only the size of *Q* ^(*i*)^ depends on *Q* in this procedure. For this reason, in our experiments, we do not explicitly generate a null distribution for each *Q*∈*C*(*S*). Rather, we generate a null distribution for sub-networks of size 2, 4, 8, 16, 32, 64. Then we interpolate the mean and standard deviation of synergistic differential expression for these distributions, to obtain a curve that characterizes the behavior of synergistic differential expression with respect to sub-network size.

### Sub-Network Classification

In order to assess the reproducibility of discovered subnetworks across different data sets and evaluate the potential of the proposed framework for feature selection in classification of CRC, we perform cross-classification experiments. In these experiments, we use the aggregate expression profiles (*e(Q)*) of crosstalker and interactor subnetworks associated with Nibbe and CAN seeds as features for classification. For this purpose, in each experiment, we select the crosstalker (or interactor) subnetworks with synergistic differential expression (ϕ(*Q*)) one standard deviation above random mean, according to a specific mRNA expression data set (e.g., GSE8671). Assume that there are *K* such subnetworks. Then, for each *k*≤K, we use the *k* subnetworks with maximum ϕ(*Q*) to train an SVM classifier on the same data set (GSE8671), using Matlab's svmtrain function. Subsequently, we use this classifier to predict the class (tumor *vs.* normal) of each sample on a different data set (e.g., GSE10950), using Matlab's svmclassify function. We evaluate the performance of the classifier using the harmonic mean of precision (selectivity) and recall (sensitivity), known as the *F*-measure, defined as
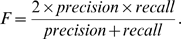
Here, *precision* is the fraction of true positives among all samples classified as tumor and *recall* is the fraction of tumor samples called accurately by the classifier among all tumor samples.

## Supporting Information

Table S1 Sub-network mutual information and classification scores. Cross-classification data are listed for all significant (MI> = 0.35) sub-networks induced by the respective seed. “CAN” refers CRC-driver gene seeds.
(0.14 MB DOC)
