# Supplementary material for: An Integrative -omics Approach to Identify Functional Sub-Networks in Human Colorectal Cancer
Source: PLoS Comput Biol. 2010 Jan 15;6(1):e1000639. doi: 10.1371/journal.pcbi.1000639 (PMC2797084; doi:10.1371/journal.pcbi.1000639)
Supplement: Table S1 — Sub-network mutual information and classification scores. Cross-classification data are listed for all significant (MI> = 0.35) sub-networks induced by the respective seed. “CAN” refers CRC-driver gene seeds. (0.14 MB DOC) [file pcbi.1000639.s001.doc]

**Supplemental Table 1. Sub-network mutual information and classification scores.** Cross-classification data are listed for all significant (MI >= 0.35) sub-networks induced by the respective seed. “CAN” refers CRC-driver gene seeds.

| **Training 10950, Test 8671** | | | | | | |
| --- | --- | --- | --- | --- | --- | --- |
| **Nibbe Crosstalkers** | |  |  |  |  |  |
| Rank | Seed | SubnetworkSize | SubnetworkMI | Precision | Recall | F-score |
| 1 | TCP1 | 11 | 0.65 | 0.97 | 0.94 | 0.95 |
| 2 | CCT2 | 10 | 0.62 | 0.94 | 0.94 | 0.94 |
| 3 | HNRPF | 20 | 0.61 | 0.94 | 0.94 | 0.94 |
| 4 | ACTG2 | 2 | 0.46 | 0.93 | 0.88 | 0.9 |
| 5 | HNRPH1 | 20 | 0.46 | 0.93 | 0.88 | 0.9 |
| 6 | SYNCRIP | 25 | 0.46 | 0.93 | 0.81 | 0.87 |
| 7 | CAPZA1 | 7 | 0.45 | 0.92 | 0.72 | 0.81 |
| 8 | TPI1 | 3 | 0.43 | 0.92 | 0.75 | 0.83 |
| 9 | SNX6 | 2 | 0.37 | 0.93 | 0.84 | 0.89 |
| 10 | CES1 | 2 | 0.36 | 0.93 | 0.81 | 0.87 |
| 11 | PGM1 | 3 | 0.35 | 0.93 | 0.84 | 0.89 |
| **Nibbe Interactors** | |  |  |  |  |  |
| 1 | HNRPF | 28 | 0.63 | 0.91 | 0.91 | 0.91 |
| 2 | HNRPH1 | 29 | 0.59 | 0.93 | 0.88 | 0.9 |
| 3 | TCP1 | 15 | 0.58 | 0.94 | 0.91 | 0.92 |
| 4 | CCT2 | 13 | 0.58 | 0.93 | 0.88 | 0.9 |
| 5 | CAPZA1 | 10 | 0.57 | 0.94 | 0.94 | 0.94 |
| 6 | HSPD1 | 40 | 0.56 | 0.94 | 0.94 | 0.94 |
| 7 | SERPINB2 | 6 | 0.55 | 0.94 | 0.94 | 0.94 |
| 8 | SNX6 | 15 | 0.54 | 0.86 | 1 | 0.93 |
| 9 | PDIA5 | 2 | 0.52 | 0.86 | 1 | 0.93 |
| 10 | HSP90AB1 | 55 | 0.49 | 0.84 | 1 | 0.91 |
| 11 | LMNA | 37 | 0.47 | 0.84 | 1 | 0.91 |
| 12 | ACTG2 | 2 | 0.46 | 0.83 | 0.94 | 0.88 |
| 13 | SYNCRIP | 36 | 0.45 | 0.84 | 0.97 | 0.9 |
| 14 | HSPA5 | 51 | 0.44 | 0.84 | 1 | 0.91 |
| 15 | ANXA3 | 7 | 0.44 | 0.84 | 1 | 0.91 |
| 16 | ALDH2 | 8 | 0.43 | 0.82 | 1 | 0.9 |
| 17 | ACTR3 | 18 | 0.43 | 0.86 | 1 | 0.93 |
| 18 | PPA1 | 7 | 0.43 | 0.84 | 1 | 0.91 |
| 19 | RUVBL2 | 26 | 0.42 | 0.89 | 1 | 0.94 |
| 20 | TALDO1 | 3 | 0.37 | 0.89 | 1 | 0.94 |
| 21 | PGM1 | 3 | 0.36 | 0.91 | 1 | 0.96 |
| **CAN Crosstalkers** | |  |  |  |  |  |
| 1 | EVL | 5 | 0.64 | 0.91 | 0.91 | 0.91 |
| 2 | EPHB6 | 2 | 0.59 | 0.96 | 0.84 | 0.9 |
| 3 | APC | 12 | 0.59 | 0.94 | 0.97 | 0.95 |
| 4 | TP53 | 2 | 0.58 | 0.94 | 1 | 0.97 |
| 5 | P2RX7 | 2 | 0.57 | 0.91 | 0.97 | 0.94 |
| 6 | PRKD1 | 2 | 0.53 | 0.91 | 1 | 0.96 |
| 7 | OBSCN | 4 | 0.47 | 1 | 0.94 | 0.97 |
| 8 | ABCA1 | 7 | 0.39 | 1 | 0.97 | 0.98 |
| 9 | ERCC6 | 3 | 0.35 | 1 | 0.94 | 0.97 |
| **CAN Interactors** | |  |  |  |  |  |
| 1 | P2RX7 | 4 | 0.59 | 0.9 | 0.56 | 0.69 |
| 2 | OBSCN | 6 | 0.54 | 0.92 | 0.72 | 0.81 |
| 3 | APC | 30 | 0.51 | 0.96 | 0.84 | 0.9 |
| 4 | GNAS | 32 | 0.44 | 0.97 | 0.88 | 0.92 |
| 5 | TP53 | 236 | 0.4 | 0.97 | 0.88 | 0.92 |

| **Training 8671 Test 10950** | | | | | | |
| --- | --- | --- | --- | --- | --- | --- |
| **Nibbe Crosstalkers** | |  |  |  |  |  |
| Rank | Seed | SubnetworkSize | SubnetworkMI | Precision | Recall | F-score |
| 1 | CCT2 | 10 | 0.66 | 0.92 | 1 | 0.96 |
| 2 | TCP1 | 11 | 0.64 | 1 | 1 | 1 |
| 3 | CAPG | 2 | 0.61 | 0.88 | 0.96 | 0.92 |
| 4 | HNRPF | 20 | 0.58 | 0.85 | 0.92 | 0.88 |
| 5 | TUBB | 52 | 0.55 | 0.88 | 0.96 | 0.92 |
| 6 | SYNCRIP | 24 | 0.49 | 0.88 | 0.92 | 0.9 |
| 7 | HNRPH1 | 20 | 0.45 | 0.92 | 0.92 | 0.92 |
| 8 | PMPCB | 3 | 0.39 | 0.88 | 0.96 | 0.92 |
| 9 | ANXA3 | 3 | 0.38 | 0.91 | 0.83 | 0.87 |
| 10 | CAPNS1 | 3 | 0.35 | 0.95 | 0.75 | 0.84 |
| **Nibbe Interactors** | |  |  |  |  |  |
| 1 | CCT2 | 13 | 0.65 | 0.88 | 0.92 | 0.9 |
| 2 | TCP1 | 15 | 0.64 | 0.92 | 0.92 | 0.92 |
| 3 | CAPG | 2 | 0.61 | 0.88 | 0.96 | 0.92 |
| 4 | TUBB | 97 | 0.56 | 0.85 | 0.96 | 0.9 |
| 5 | RUVBL2 | 29 | 0.54 | 0.88 | 0.96 | 0.92 |
| 6 | HNRPH1 | 29 | 0.53 | 0.85 | 0.96 | 0.9 |
| 7 | HNRPF | 28 | 0.52 | 0.85 | 0.96 | 0.9 |
| 8 | HSPD1 | 41 | 0.5 | 0.85 | 0.96 | 0.9 |
| 9 | SELENBP1 | 6 | 0.49 | 0.88 | 0.96 | 0.92 |
| 10 | LMNA | 38 | 0.48 | 0.88 | 0.96 | 0.92 |
| 11 | TAGLN | 3 | 0.44 | 0.92 | 0.96 | 0.94 |
| 12 | ANXA3 | 7 | 0.43 | 0.95 | 0.83 | 0.89 |
| 13 | SYNCRIP | 34 | 0.43 | 0.95 | 0.83 | 0.89 |
| 14 | PMPCB | 4 | 0.42 | 0.91 | 0.83 | 0.87 |
| 15 | COMT | 5 | 0.41 | 0.96 | 0.92 | 0.94 |
| **CAN Crosstalkers** | |  |  |  |  |  |
| 1 | APC | 12 | 0.51 | 0.96 | 0.92 | 0.94 |
| 2 | EVL | 5 | 0.44 | 1 | 0.96 | 0.98 |
| 3 | ABCA1 | 7 | 0.44 | 1 | 1 | 1 |
| 4 | PRKD1 | 2 | 0.38 | 1 | 1 | 1 |
| **CAN Interactors** | |  |  |  |  |  |
| 1 | TP53 | 244 | 0.57 | 0.79 | 0.92 | 0.85 |
| 2 | GNAS | 32 | 0.44 | 0.92 | 1 | 0.96 |
| 3 | ERCC6 | 11 | 0.44 | 0.96 | 1 | 0.98 |
